# Ukraine and singular optics

**DOI:** 10.1515/nanoph-2022-0674

**Published:** 2023-02-10

**Authors:** Michael Berry

**Affiliations:** H H Wills Physics Laboratory, Tyndall Avenue, Bristol BS8 1TL, UK

**Keywords:** caustics, interference, polarisation, waves

## Abstract

The mostly theoretical viewpoint, in which light is understood in terms of its singularities – caustics, phase vortices and lines of circular and linear polarisation – was developed in Bristol in the 1970s and 1980s. Starting in the 1990s, research by physicists in Ukraine, principally by Marat Soskin, stimulated the development of ‘singular optics’ as a thriving area of experimental and theoretical physics worldwide.

My entanglement with Ukraine began before I was born. In 1906, my father’s parents arrived in London from Odessa. By the 1950s, my grandfather had largely forgotten Russian and spoke to me in what I can only describe as ‘affectionate Yidglish’. His one-word explanation of why they left: “Pogroms”. Perhaps these were exacerbated by Russia’s defeat in the war with Japan, and the abortive revolution later fictionalised on film in Eisenstein’s *Battleship Potemkin.* Family mythology claimed that my grandfather was born in Azerbaijan, making me one-quarter Asian; I have no way of confirming this.

Enough about roots. In today’s world, there is too much emphasis on roots. I prefer leaves, branches, and flowers – also fruits, in particular science, where Ukraine had a quite unexpected influence on my life as a physicist.

I need to backtrack. Before the connection to Ukraine, in the 1970s, in Bristol, with involvement from my senior colleague John Nye, I had been developing a way of understanding light – and wave physics generally – in terms of *singularities*.

This started with geometrical optics, i.e. ray physics – appropriately enough, since this is the oldest branch of optics. The ray singularities are *caustics* – typically, lines in 2D and surfaces in 3D, on which rays of light are focused. They are the envelopes of families of rays. I had come to understand the significance of caustics in the 1960s, while studying semiclassical quantum physics, where caustics are the singularities of families of the underlying classical paths. Then I realised that similar phenomena occur in geometrical optics, with the attractive feature that it is possible to see the singularities with our unaided eyes, as places where the light is brightest. A hugely important input to these studies that I learned in the 1970s was the then new mathematics of catastrophe theory. This classifies the shapes of caustics that are stable under perturbation – *natural* caustics, contrasting with the focal points and associated aberrations in optical technology such as lens-making. Everyone has seen natural caustics as the cusps in coffee cups, as rainbows and as the dancing bright lines of focused sunlight on the bottoms of swimming-pools.

The development of ‘catastrophe optics’ was enriched by the discovery that each type of ray caustic is decorated by a characteristic wave pattern: its ‘diffraction catastrophe’ [1]. But the deepest penetration into wave optics was the discovery of its singularities at the next level down: the identification, with Nye, of *phase singularities* as typical features of general wave fields [2]. We called them *wave dislocations*, because of their morphological similarity to the defects in crystals that Nye had been studying. They are also *nodal points and lines*, because the wave intensity vanishes where the phase is singular. Nowadays they are often called *phase vortices*, because the optical energy flows around them. We emphasised that ‘typical features’ has the precise meaning that the singularities are stable under perturbation, unlike the ‘dark fringes’ (lines in 2D, surfaces in 3D) envisaged in traditional optics, which are special cases, usually unstable.

We realised that phase singularities are, in a precise sense, complementary to caustics. Caustics are coarse features, emerging on scales of observation much larger than the wavelength. Phase singularities, on the other hand, are sub-wavelength structures, detectable on observation scales where caustics lose their prominence by being spread by diffraction.

Phase singularities are features in scalar wave optics. The completion of our conceptual understanding of light in terms of its singularities was the discovery in the 1980s, by Nye and his student Jo Hajnal, of the analogous *polarisation singularities* in the optical electromagnetic vector fields [3]. These are the C lines, on which the polarisation is purely circular, and the L lines on which polarisation is purely linear.

This was the ‘threefold way’ of understanding light in terms of its singularities: ray caustics, wave vortices of scalar light and polarisation singularities of vector light [4, 5]. Although there had been observations and experiments at all three levels, this detailed exploration of the underlying concepts was largely theoretical. Our way of thinking about waves had been largely ignored by other physicists.

Such was the situation when Marat Soskin visited us in Bristol in 1996. We were surprised to learn that for several years he had been carrying out experiments in Kiev, to create and manipulate phase singularities [6]. He had learned about our work, and introduced the term ‘singular optics’ to describe this way of studying light. Soskin’s work exploring phase singularities was important, not only in itself but because it triggered an explosion of research in Ukraine, transforming the study of optical vortices into a science where theory and experiment evolved in close synchrony [7–9].

As I have described elsewhere, Soskin’s visit stimulated my return to the study of wave singularities, to explore many new theoretical aspects arising directly and indirectly by the experiments in Ukraine [10]. When Mark Dennis arrived as my student in 1998, we developed much of this theoretical work together: in particular, calculations of the statistics of phase and polarisation singularities [11, 12], and discovering that in three dimensions wave vortex lines can be linked and knotted [13, 14]; the topological studies were later greatly extended by Dennis.

Soskin invited me to a memorable meeting in Crimea in 1997, where I learned about the extensive studies of optical vortices by Ukrainian physicists. These were followed by two further visits to Crimea with Dennis, and a meeting in Kiev where we were joined by Nye. On one of these trips, I was also able to explore my ancestral city of Odessa. After the Russian occupation of Crimea, international meetings on singular optics continued in the relative safety of Chernivtsy in the west of Ukraine.

When Soskin learned about Nye’s polarisation singularities, he extended his investigations to study them too [15], and expanded his interpretation of singular optics to include them. So, two of the three branches of singular optics – though not yet the third, namely caustics – have been extensively studied by scientists in Ukraine. Although it is potentially invidious to mention more individuals, I also salute the following scientists, working across Ukraine, who have, along with others, made important contributions to our subject: Oleg Angelsky in Chernivtsi, Aleksandr Bekshaev in Odessa, Alexander Volyar in Simferopol and Mikhail Vasnetsov in Kiev.

Why has Ukraine been so significant and influential in the development of our understanding of phase and polarisation singularities? I can speculate. These studies require theoretical depth, technical virtuosity and experimental ingenuity, but not expensive apparatus. In post-Soviet Ukraine, high-level theoretical physics researchers, and skilled experiment lists, have continued to flourish, but funding for science has been scarce. Therefore it was natural for singular optics to emerge as a successful area of research in Ukraine.

My connections with Ukraine continue. In 2021 I was honoured by being elected as a Foreign Member of the National Academy of Sciences of Ukraine.

In this special issue of *Nanophotonics*, I have emphasised the central role of Ukraine in developing singular optics and in stimulating worldwide studies of this area of physics. But in this dark time for Ukraine (I write in October 2022), the worldwide aspect must be emphasised: science transcends national boundaries. I cannot improve on the beautiful words of the chemist Humphrey Davy from 1807, when he visited France during the war with England:

… the two countries are at war, [but] the men of science are not. That would, indeed, be a civil war of the worst description; we should rather, through the instrumentality of men of science, soften the asperities of national hostility.
